# Quality of life, hearing results, patient satisfaction and postoperative complications of day-case versus inpatient unilateral cochlear implantation in adults: a randomized controlled, equivalence trial

**DOI:** 10.1007/s00405-023-08352-8

**Published:** 2024-01-05

**Authors:** Laura S. M. Derks, Adriana. L. Smit, Hans G. X. M. Thomeer, Vedat Topsakal, Wilko Grolman, Robert J. Stokroos, Inge Wegner

**Affiliations:** 1 Department of Otorhinolaryngology – Head and Neck Surgery, University Medical Center Utrecht, G05.129, Heidelberglaan 100, 3584 CX Utrecht, The Netherlands; 2 Brain Center Rudolf Magnus, University Medical Center Utrecht, Utrecht, The Netherlands; 3 University Department Otorhinolaryngology, Head & Neck Surgery, Antwerp University Hospital, Antwerp, Belgium; 4 Faculty of Medicine and Health Sciences, Antwerp University, Antwerp, Belgium; 5 Jean Causse Ear Clinic, Traverse de Béziers, Colombiers, France; 6 Department of Otorhinolaryngology – Head and Neck Surgery, University Medical Center Groningen, Groningen, The Netherlands

**Keywords:** Cochlear implantation, Day-case, Inpatient, Quality of life, Patient satisfaction, Admission

## Abstract

**Objective:**

To investigate the hypothesis that day-case cochlear implantation is associated with equal quality of life, hearing benefits and complications rates, compared to inpatient cochlear implantation.

**Study design:**

A single-center, non-blinded, randomized controlled, equivalence trial in a tertiary referral center.

**Methods:**

Thirty adult patients with post-lingual bilateral sensorineural hearing loss eligible for unilateral cochlear implantation surgery were randomly assigned to either the day-case or inpatient treatment group. The effect on general quality of life, patient satisfaction, (subjective) hearing improvement, postoperative complications and causes of crossover and/or readmission were assessed using questionnaires, auditory evaluations and patients’ charts over a follow-up period of 1 year.

**Results:**

Overall quality of life measured by the HUI3 was equal between the day-case (*n* = 14) and inpatient group (*n* = 14). The overall patients’ satisfaction showed a slight favor towards an inpatient approach. There was no significant difference in the subjective and objective hearing improvement between both treatment groups. During the 1-year follow-up period no major complications occurred. Minor complications occurred intraoperatively in three day-case patients resulting in three out of nine admissions of day-case patients. Other causes of admission of day-case patients were nausea and vomiting (*n* = 1), drowsiness (*n* = 1), late scheduled surgery (*n* = 2), social reasons (*n* = 1), or due to an unclear reason (*n* = 1). No patients required readmission.

**Conclusion:**

We found equal outcomes of QoL, patient satisfaction, objective, and subjective hearing outcomes between day-case and inpatient unilateral cochlear implantation. Nine out of 14 day-case patients were admitted for at least one night postoperatively (crossover). No major complications occurred in both groups. A day-case approach seems feasible when using specific patient selection, surgical planning and the preoperative provision of patient information into account. Besides this, the familiarity with a day-case approach of both patient and the surgical team can increase the feasibility of day-case surgery.

**Level of evidence:**

1.

**Supplementary Information:**

The online version contains supplementary material available at 10.1007/s00405-023-08352-8.

## Introduction

Cochlear implantation is a common procedure in the treatment of severe to profound bilateral sensorineural hearing loss (SNHL) in children and adults [1–4]. Like many (otologic) surgical procedures, cochlear implantation is increasingly being performed as a day-case procedure in the Netherlands and other countries (e.g., USA, Canada, United Kingdom) [5–10]. Besides the (potential) financial benefit of performing surgery as a day-case procedure, it is also associated with shorter waiting time for surgery and reduced risk of hospital-acquired infection [11]. Moreover, as a result of a more rapid social and emotional rehabilitation compared to overnight stay, patients might prefer day-case surgery resulting in an increased quality of life (QoL) [9, 10, 12]. Advances in anesthetic techniques increase the feasibility of a day-case approach, including local infiltration of analgesics, the use of less and shorter-acting intraoperative anesthetic drugs, especially morphine, and inhalants and better prophylaxis for postoperative nausea and vomiting (PONV) [13, 14]. Moreover, due to the elective nature of the procedure, preoperative counselling and evaluation of the presence of comorbidities is possible. Furthermore, the procedure is associated with low direct postoperative complication rates: 1–9% for (transient) vertigo, 1–3% for tinnitus, 1–3% for postoperative bleeding or hematoma and < 1% for facial nerve injury [15–18]. However, reports on day-case cochlear implantation supporting equal outcomes compared to an inpatient approach are scarce and mostly describe pediatric day-cases [5–9]. Only one of these studies compared the outcomes of day-case to inpatient surgery, finding no advantages of inpatient surgery compared to a day-case approach [10].

The aim of this randomized controlled trial is to investigate the hypothesis that day-case cochlear implantation in adults is associated with an at least equal QoL and with equal hearing benefits, patient satisfaction and complications rates, compared to inpatient cochlear implantation.

## Materials and methods

This article is based on data acquired in a single-center, non-blinded, randomized controlled trial, approved by the Institutional Review Board of the University Medical Center Utrecht (NL45590.041.13). This study was registered in the Netherlands Trial Register (www.trialregister.nl; NTR4464, March 13th 2014). The complete study protocol was published in October 2016 [19]. There were no changes to the study methods or outcomes after the study commenced. The data of this randomized controlled trial are reported according to the CONSORT Statement [20–22].

### Study population

**Fig. 1:**
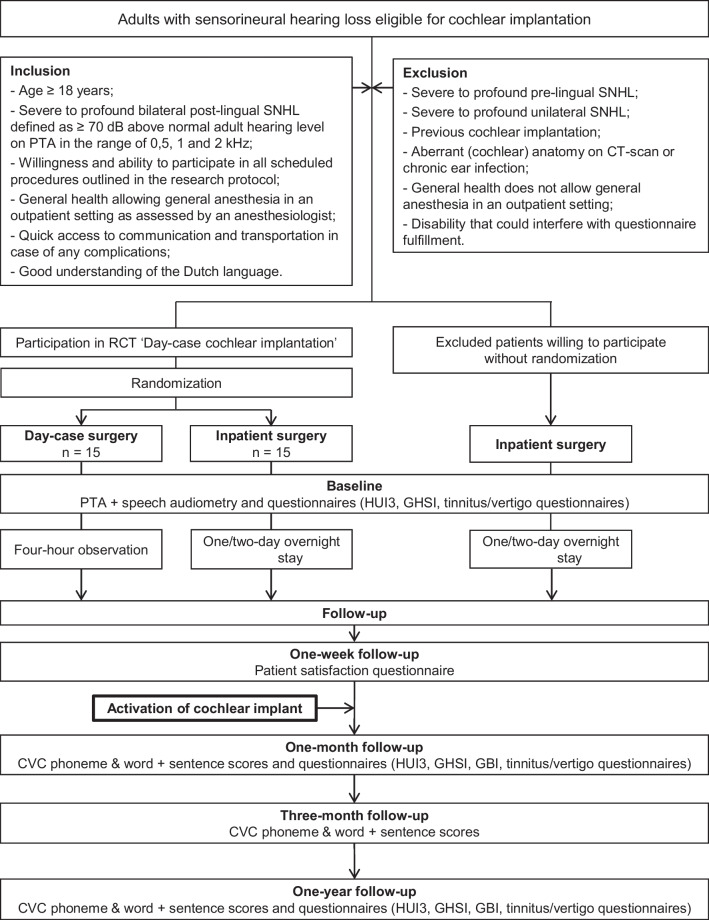
Flow chart study protocol. *CVC* consonant–vowel-consonant, *dB* decibel, *GBI* Glasgow Benefit Inventory, *GHSI* Glasgow Health Status Inventory, *HUI3* Health Utilities Index –Mark 3, *n* number of patients, *PTA* pure-tone audiometry, *SNHL* sensorineural hearing loss

Patients undergoing a unilateral cochlear implantation were eligible to participate if they met all inclusion criteria (Fig. 1).

### Samples size calculation

To establish equivalence in general QoL with a margin of equivalence of 0.15 points with a standard deviation (SD) of 0.15 on the Health Utilities Index – Mark 3 (HUI3) between the day-case and inpatient group with an alpha of 0.05 and power of 80%, 14 participants per group were needed. To anticipate 10% withdrawal of participants, a total of 30 participants were recruited.

### Randomization, blinding and treatment allocation

Patients were enrolled by one of two researchers (authors LD and IW). After inclusion, patients were allocated to either the conventional group (inpatient surgery) or the day-case group using a web-based randomization program (Julius Center, UMC Utrecht, Utrecht, the Netherlands). Patients were randomly allocated into two groups with stratification for age. Block randomization was used with an allocation ratio 1:1. The randomization chart, including block size, was established by an independent data manager before the start of the study. Consequently, treatment allocation sequence was concealed for patients, care providers and researchers. Blinding of the involved was not possible because patients and care providers would be aware of the surgical setting and hospital stay. Crossover was defined as hospital admission of a day-case patient for at least one night, or, for the inpatient group as discharge on the day of surgery. Crossover between groups and causes thereof were assessed using patients’ charts. Readmission was defined as admission after initial discharge. In case of crossover, patients were asked to complete their follow-up, and analyses were carried out on an intention-to-treat basis.

### Baseline

Baseline characteristics were assessed using patients’ charts. Preoperatively patients were asked to fulfill the HUI3 [23, 24] and Glasgow Health Status Inventory (GHSI) [25] questionnaires as a baseline measurement. If patients suffered from tinnitus and/or vertigo preoperatively, they were also asked to complete tinnitus and vertigo questionnaires. These included the Tinnitus Handicap Index (THI) [26, 27], Tinnitus Questionnaire (TQ) [28, 29], Dizziness Handicap Inventory (DHI) [30, 31] and the Utrecht Burden Questionnaire for tinnitus (UBQT) and vertigo (UBQV) (Appendix 1).

### Intervention

All surgical procedures were performed under general anesthesia by one of three surgeons (authors AS, HT and VT) in the same tertiary referral center (University Medical Center Utrecht). Patients allocated to the inpatient group were admitted one day before or on the day of surgery and were discharged one to two days after surgery. Patients allocated to the day-case group were admitted to the ward one day before or on the day of surgery and were discharged the day of surgery. If postoperatively patients were judged as not being physically capable of same-day discharge or if the surgeon did not support same-day discharge, patients would stay overnight.

### Primary outcome measure

The primary outcome was the general QoL measured by the HUI3 at 3 weeks and 1 year postoperatively. The HUI3 is a 15-item questionnaire that measures general health by evaluating eight domains: vision, hearing, speech, ambulation, dexterity, cognition, emotion and pain. The outcome is a multi-attribute health status resulting in a utility score between -0.36 and 1.00, corresponding to a state worse than death and perfect health, respectively [23, 24]. For this study only the total utility score and hearing subscore outcomes were used. To assess equivalence, a margin in the between-group mean difference in total HUI3 scores of ± 0.15 points was considered as an equal outcome [24].

### Secondary outcome measures

The secondary outcome measures included patient satisfaction, (subjective) hearing improvement, and postoperative complications (including tinnitus and vertigo). Patient satisfaction was evaluated one week postoperatively using the Utrecht Patient Satisfaction Survey (UPSS) (Appendix 2).

Subjective hearing benefit was evaluated at three weeks (cochlear implant not yet activated) and 1 year (cochlear implant activated) postoperatively using the GHSI and Glasgow Benefit Inventory (GBI) [32, 33]. For both questionnaires the outcome of the total and three subscores (general, social support and physical health) were reported. Objective hearing results were assessed at 1 month, 3 months and 1 year postoperatively using a set of Dutch words with a consonant–vowel-consonant structure (CVC) in a free field setting and a test whereby (Dutch) sentences are asked to be repeated. The outcome measures are the percentage of correctly repeated words (CVC score) and the percentage of correctly repeated complete sentences (STS), words (WS) and phonemes (PS). The measurements were performed under optimal conditions, with hearing aids preoperatively and postoperatively with the cochlear implant, with or without a hearing aid in the contralateral ear as used in daily life situations.

Postoperative complications were prospectively registered in the patients’ chart and classified according to the Hoffman and Cohen’s criteria [34]. Complications were considered major if hospitalization or revision surgery were required and minor if they resolved spontaneously or if only medication was required. Differentiation was made between perioperative (during surgery), directly postoperative, early (within 1 month after surgery) and late (within 1 year after surgery) complications. The presence and burden of tinnitus and vertigo were assessed at 3 weeks and 1 year postoperatively using the THI, TQ and UBQT for tinnitus, and the DHI and UBQV for vertigo. For the THI, TQ and DHI the total score, subscores and severity score were reported.

### Statistical analyses

Means and SDs or percentages were calculated for all baseline characteristics per group. Differences in the baseline were analyzed using the Mann–Whitney *U* Test for continuous variables and the Fisher’s exact test for categorical variables. Non-parametric tests were used as none of the outcome data were normally distributed. Normality was analyzed using mean, median, histogram and boxplots. A *p* value < 0.05 (2-tailed) was considered statistically significant. The primary and secondary outcome data were both continuous and categorical. Means, SDs and percentages were calculated. Between-group mean differences, rate differences and rate ratios with 95% confidence intervals (CIs) were calculated. The Mann–Whitney *U* Test for continuous variables and the Fisher’s exact test for categorical variables were used for further analyses of between-group differences.

Missing values were handled using multiple imputation and all analyses were performed on an intention-to-treat basis. Data of questionnaires that were returned empty were not imputed. All analyses were performed using SPSS version 25.0 (SPSS Inc., Chicago, IL).

## Results

### Patient flow

**Fig. 2:**
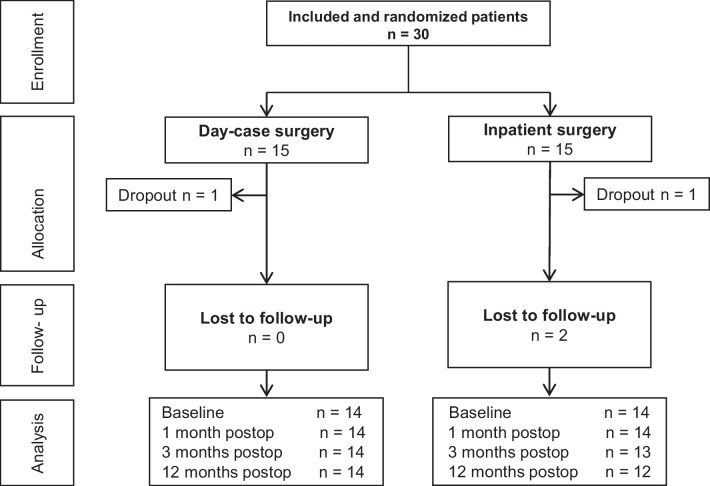
Flow chart included patients. *n* number of patients

A total of 30 patients were included from April 2014 to April 2019 (Fig. 2). Follow-up took place from April 2014 to July 2020. 28 (93%) cases received treatment. Two patients dropped out after treatment allocation and before receiving treatment: one in each randomization group. In one patient the surgery was cancelled because of severe cardiologic comorbidity and one patient requested cancellation of the operation. The two dropouts were excluded from all analyses. 25 patients (89%) were analyzed for the primary outcome QoL measured by the HUI3 at 3 weeks postoperatively: 14 patients in the inpatient group and 11 in the day-case group. 22 patients (79%) were analyzed for the primary outcome at 1 year postoperatively: 11 in each group. Six patients did not complete the 1-year follow-up period, five patients due to an unknown cause and one patient died of a comorbidity unrelated to the surgery.

### Baseline characteristics

There were no significant between-group differences in the baseline characteristics (Table 1).

**Table 1:** Patients characteristics at baseline

	Inpatient (*n* = 14)	Day-case (*n* = 14)	Mean difference
Sex (male:female)	5:9	6:8	^b^
Age at surgery (mean (SD) in years)	59 (35–75)	62 (39 to 81)	– 2 (– 12 to 8)^a^
Cause of deafness (*n* (%)), operated side			
Congenital SNHL	2 (14)		^b^
Hereditary hearing loss	4 (28)	2 (14)	
Sudden deafness	2 (14)	1 (7)	
Meniere’s disease		3 (21)	
Otosclerosis	3 (21)		
Meningitis		2 (14)	
Toxoplasmosis		1 (7)	
Progressive hearing loss	1 (7)	3 (21)	
Other	1 (7)	2 (14)	
Unknown	1 (7)		
Perioperative characteristics			
Side of surgery (left:right)	4:10	3:11	^b^
Implant, brand (*n* (%))			
Cochlear	10 (71)	7 (50)	^b^
MED-EL		3 (21)	
Advanced Bionics	4 (29)	3 (21)	
Oticon		1 (7)	
Surgical approach (*n* (%))			
Suprameatal approach	7 (50)	5 (36)	^b^
Post tympanotomy	7 (50)	9 (64)	
Insertion site of electrode (*n* (%))			
Round window	6 (43)	7 (50)	^b^
Cochleostoma	8 (57)	7 (50)	
Insertion of electrode (*n* (%))			
Complete	14 (100)	13 (93)	^b^
Incomplete		1 (7)	
Duration of surgery (mean (SD) in minutes)	121 (27)	125 (30)	– 4 (– 26 to 18)^a^

None of the differences in the baseline characteristics between groups were statistically significant

*N *number of patients, *SD* standard deviation, *SNHL* sensorineural hearing loss

^a^Independent-samples Mann–Whitney *U* Test

^b^Fisher’s exact test (2-sided)

### Quality of life

**Fig. 3:**
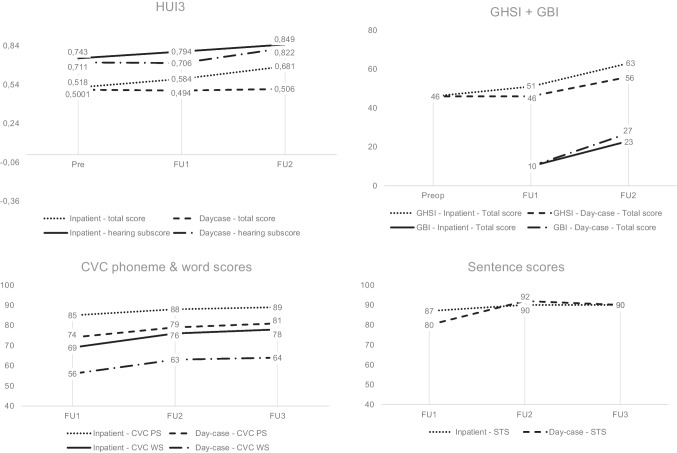
Outcome measures. *CVC* consonant–vowel-consonant structure, *FU* follow-up, *GBI* Glasgow Benefit Inventory, *GHSI* Glasgow Health Status Inventory, *HUI3* Health Utilities Index –Mark 3, *PS* phoneme scores, *STS* sentence scores, *WS* word scores

The overall QoL, measured with the HUI3, at 3 weeks postoperatively was equal in both groups (mean difference = 0.09 points) (Table 2: HUI3; Fig. 3: HUI3). The total score differed 0.17 at 1 year postoperatively between groups. The hearing subscore differed 0.08 points at three weeks (statistically significant) and 0.03 points at 1-year follow-up.

**Table 2:** Outcomes of used questionnaires

	Inpatient			Day-case			Difference (95% CI)		
	Pre-operative	3 weeks postop	1 year postop	Pre-operative	3 weeks postop	1 year postop	Preoperative	3 weeks postop	1 year postop
HUI3 (mean (SD))									
Total score	0.52 (0.20)	0.58 (0.19)	0.68 (0.18)	0.50 (0.13)	0.49 (0.16)	0.51 (0.27)	0.02 (– 0.10 to 0.14)^a^	0.09 (– 0.05 to 0.23)^a^	0.17 (– 0.02 to 0.37)^a^
Missing (*n*)	0	0	3	0	3	4	0.03 (– 0.02 to 0.09)^a^	**0.08 (0.02 to 0.15)**^a^	0.03 (– 0.03 to 0.09)^a^
Hearing subscore	0.74 (0.08)	0.79 (0.07)	0.85 (0.07)	0.71 (0.07)	0.71 (0.09)	0.82 (0.08)			
Missing (*n*)	0	0	3	0	3	3			
GHSI (mean (SD))	*n* = 14	*n* = 11	*n* = 6	*n* = 14	*n* = 7	*n* = 5			
Total score	46 (12)	51 (12)	63 (10)	46 (15)	46 (12)	56 (18)	0 (– 8 to 9)^a^	5 (– 6 to 16)^a^	8 (– 9 to 25)^a^
General subscore	32 (16)	38 (17)	56 (14)	38 (18)	43 (13)	53 (18)	– 6 (– 17 to 6)^a^	– 5 (– 19 to 9)^a^	3 (– 16 to 21)^a^
Social support subscore	89 (7)	86 (8)	75 (14)	76 (21)	65 (30)	70 (32)	13 (1 to 24)^a^	20 (2 to 39)^a^	5 (– 23 to 33)^a^
Physical health subscore	60 (28)	67 (20)	83 (11)	47 (17)	39 (12)	53 (22)	13 (– 4 to 30)^a^	**28 (12 to 44)**^a^	**30 (10 to 50)**^a^
GBI (mean (SD))		*n* = 12	*n* = 11		*n* = 10	*n* = 11			
Total score		10 (15)	23 (21)		11 (16)	27 (20)		– 1 (– 13 to 11)^a^	– 4 (– 20 to 12)^a^
General subscore		11 (22)	32 (33)		14 (22)	41 (23)		– 2 (– 20 to16)^a^	– 9 (– 32 to 14)^a^
Social support subscore		11 (15)	9 (23)		13 (22)	8 (11)		– 2 (– 18 to 13)^a^	2 (– 14 to 17)^a^
Physical health subscore		3 (10)	4 (14)		– 2 (5)	– 8 (22)		5 (– 2 to 12)^a^	11 (– 5 to 27)^a^
THI (mean (SD))	*n* = 6	*n* = 5	*n* = 4	*n* = 12	*n* = 9	*n* = 8			
Total score	34 (25)	37 (23)	37 (11)	13 (8)	15 (16)	9 (7)	21 (6 to 37)^a^	22 (2 to 42)^a^	**28 (17 to 40)**^a^
Emotional subscale	8 (6)	8 (6)	10 (7)	2 (2)	3 (4)	1 (1)	6 (2 to 10)^a^	5 (0 to 10)^a^	9 (4 to 13)^a^
Functional subscale	21 (15)	23 (13)	22 (6)	9 (5)	10 (10)	7 (5)	12 (3 to 21)^a^	13 (1 to 25)^a^	**15 (9 to 20)**^a^
Catastrophic subscale	6 (4)	6 (5)	6 (2)	3 (3)	2 (3)	1 (2)	3 (0 to 6)^a^	4 (0 to 8)^a^	**5 (2 to 8)**^a^
THI; severity (%)	*n* = 6	*n* = 5	*n* = 4	*n* = 12	*n* = 9	*n* = 8			
Slight handicap	33	20	0	58	67	88	^**b**^	^b^	^**b**^
Mild handicap	0	20	50	42	22	12			
Moderate handicap	67	40	50	0	11	0			
Severe handicap	0	20	0	0	0	0			
TQ (mean (SD))	*n* = 7	*n* = 6	*n* = 4	*n* = 11	*n* = 9	*n* = 8			
Total score	33 (17)	35 (21)	41 (5)	18 (10)	12 (8)	12 (8)	15 (2 to 29)^a^	22 (8 to 37)^a^	**29 (21 to 37)**^a^
Emotional distress	12 (7)	12 (8)	15 (4)	5 (4)	4 (2)	4 (3)	6 (1 to 12)^a^	8 (3 to 13)^a^	**11 (7 to 15)**^a^
Auditory perceptual difficulties	8 (6)	9 (6)	9 (4)	4 (4)	3 (3)	2 (2)	4 (– 1 to 9)^a^	6 (2 to 10)^a^	**7 (4 to 10)**^a^
Intrusiveness	7 (3)	7 (4)	9 (1)	4 (2)	3 (2)	4 (2)	3 (0 to 5)^a^	4 (1 to 7)^a^	**5 (3 to 7)**^a^
Sleep disturbances	2 (2)	2 (2)	3 (4)	1 (3)	0 (1)	0 (1)	0 (– 2 to 3)^a^	2 (0 to 4)^a^	3 (0 to 5)^a^
Somatic complaints	2 (2)	2 (3)	4 (2)	1 (2)	0 (1)	0 (1)	1 (– 1 to 3)^a^	2 (0 to 4)^a^	**3 (1 to 5)**^a^
UBQ Tinnitus	*n* = 8	*n* = 7	*n* = 5	*n* = 10	*n* = 8	*n* = 7			
VAS (mean (SD))	6 (3)	6 (3)	8 (1)	4 (2)	4 (3)	4 (2)	2 (– 1 to 4)^a^	2 (– 1 to 5)^a^	**4 (2 to 6)**^a^
DHI (mean (SD))	*n* = 4	*n* = 4	*n* = 2	*n* = 5	*n* = 3	*n* = 1			
Total score	29 (4)	29 (3)	30 (11)	29 (8)	29 (5)	28 (–)	– 1 (– 11 to 9)^a^	0 (– 7 to 8)^a^	2 (– 174 to 178)^a^
Physical subscore	9 (2)	9 (2)	6 (3)	8 (3)	8 (4)	10 (–)	1 (– 3 to 5)^a^	1 (– 4 to 7)^a^	– 4 (– 48 to 40)^a^
Functional subscore	10 (2)	11 (2)	12 (4)	10 (3)	11 (3)	7 (3)	0 (– 4 to 4)^a^	– 1 (– 5 to 3)^a^	5 (– 9 to 18)^a^
Emotional subscore	10 (0)	10 (1)	13 (5)	11 (4)	10 (1)	9 (0)	– 1 (– 6 to 3)^a^	0 (– 1 to 1)^a^	4 (– 12 to 19)^a^
DHI; severity (%)	*n* = 4	*n* = 4	*n* = 2	*n* = 5	*n* = 3	*n* = 1			
Mild handicap	100	100	50	80	100	100	^b^	NC	^b^
Moderate handicap	0	0	50	20	0	0			
Severe handicap	0	0	0	0	0	0			
UBQ Vertigo	*n* = 6	*n* = 5	*n* = 6	*n* = 5	*n* = 5	*n* = 6			
VAS (mean (SD))	2 (1)	2 (1)	3 (1)	2 (1)	1 (2)	3 (2)	0 (– 1 to 2)^a^	1 (– 1 to 3)^a^	0 (– 2 to 1)^a^

Differences printed in bold were statistically significant (*p* < 0.05)

*HUI3* Health Utility Index 3, *GHSI* Glasgow Health Status Inventory, *GBI* Glasgow Benefit Inventory, *THI* Tinnitus Handicap Inventory, *TQ* Tinnitus Questionnaire, *UBQT* Utrecht Burden Questionnaire Tinnitus, *DHI* Dizziness Handicap Inventory, *TBQV* Utrecht Burden Questionnaire Vertigo, *n* number of patients, *SD* standard deviation, *CI* confidence interval, *NC* not computable

^a^Independent-Samples Mann–Whitney U Test

^b^Fisher’s exact test

### Patient satisfaction

The overall mean patient satisfaction score of the first postoperative night on a scale of 0 (very easy) to 10 (very difficult) was 3.5 (SD 2.3) in the inpatient group and 4.9 (SD 2.3) in the day-case group; the mean difference of – 1.4 was not statistically significant. Of the patients allocated to the day-case group, 75% would undergo the surgery in day-case again. 50% would have preferred to have spent the night in the hospital in hindsight, whereas only 7% of patients in the inpatient group would have preferred to have spent the night at home. Appendix 3 shows the outcomes of the complete UPSS.

### Subjective and objective hearing improvement

There was no significant difference in the total GHSI and GBI scores between both treatment groups at both follow-up moments. The only subscore with a statistically significant difference between groups was the physical health subscore of the GHSI, showing lower scores for the day-case group at three weeks and 1 year postoperatively: 67 versus 39 at three weeks and 83 versus 53 at 1 year (Table 2; Fig. 3).

The mean CVC phoneme score did not differ significantly between the inpatient and day-case group at both follow-up moments. The mean CVC word scores were statistically significantly lower at 1 month postoperatively in the day-case group (69% versus 57%) and were not statistically significantly different at 3 months and 1 year postoperatively. The mean sentence test scores were not significantly different between both groups (Table 3; Fig. 3).

**Table 3:** Postoperative objective hearing results

	Inpatient (*n* = 14)	Day-case (*n* = 14)	Mean difference (95% CI)
CVC phoneme score (mean % (SD))^a^			
1 month postop	85 (13)	74 (21)	10 (– 3 to 23)^c^
3 months postop	88 (13)	79 (22)	9 (– 4 to 22)^c^
1 year postop	89 (9)	81 (20)	8 (– 3 to 19)^c^
CVC word score (mean % (SD))^a^			
1 month postop	69 (19)	57 (25)	13 (– 4 to 29)^c^
3 months postop	76 (20)	63 (30)	12 (– 6 to 31)^c^
1 year postop	78 (16)	66 (28)	12 (– 4 to 28)^c^
Sentence test score (mean % (SD))^b^			
1 month postop	87 (23)	80 (28)	7 (– 12 to 26)^c^
3 months postop	90 (15)	92 (13)	– 1 (– 12 to 9)^c^
1 year postop	90 (16)	90 (21)	1 (– 12 to 14)^c^

Differences printed in bold were statistically significant (*p* < 0.05)

*CI* confidence interval, *CVC* Consonant–vowel-consonant, *SD* standard deviation

^a^CVC phoneme and word score at 65 dB SPL in quiet

^b^Sentence test score: percentage of correctly replied sentences

^c^Independent-Samples Mann–Whitney U Test

### Postoperative complications, tinnitus and vertigo

No major complications occurred during our 1-year follow-up period (Table 4). Intraoperative minor complications occurred in three day-case patients. Direct postoperative complications were nausea without (*n* = 1 day-case) and with (*n* = 2 inpatient; *n* = 2 day-case) vomiting. Early postoperative complications were vertigo (*n* = 5 inpatient; *n* = 3 day-case), tinnitus (*n* = 4 inpatient; *n* = 5 day-case) and wound infection (*n* = 1 day-case). Late complications were vertigo (*n* = 5 inpatient; *n* = 4 day-case), tinnitus (*n* = 5 inpatient; *n* = 6 day-case), altered position of the implant (*n* = 1 inpatient; *n* = 1 day-case) and facial nerve stimulation (*n* = 1 day-case). Altered taste directly postoperative was reported by three (day-case) patients, one (inpatient) patient reported an altered taste 1 year after surgery.

**Table 4:** Complications

	Inpatient (*n* = 14)	Day-case (*n* = 14)
Intraoperative complications (*n* (%))		
None	14 (100)	11 (79)
Gusher		1 (7)
Allergic reaction		1 (7)
Hypertension		1 (7)
Direct postoperative complications (*n* (%))		
None	12 (86)	11 (79)
Nausea		1 (7)
Nausea and vomiting	2 (14)	2 (14)
Early postoperative complications (*n* (%)), < 1 month		
None	6 (43)	1 (7)
Vertigo	5 (36)	3 (21)
Tinnitus	4 (29)	5 (36)
Wound infection		1 (7)
Altered taste (*n* = 11, missing *n* = 3)		3 (27)
Late postoperative complications (*n* (%)), up to 1 year	*n* = 13	*n* = 11
None	4 (31)	2 (18)
Vertigo	5 (38)	4 (36)
Tinnitus	5 (38)	6 (55)
Altered position implant	1 (8)	1 (9)
Facial nerve stimulation		1 (9)
Altered taste (*n* = 11, missing *n* = 3)	1 (9)	

All complications were classified as ‘minor’. None of the between group differences were statistically significant (*p* < 0.05) using the Fisher’s Exact Test (2-sided)

*n* number of patients.

*Tinnitus* –The mean total THI scores and functional and catastrophic THI subscores were significantly higher in the inpatient group at 1-year follow-up. The mean total and all subscores, with exception of the sleep disturbance subscore, of the TQ were significantly higher in de inpatient group at 1-year follow-up. The mean VAS score was statistically significantly higher in the inpatient group.

*Vertigo –* There were no statistically significant differences in the outcomes of the DHI, UBQV and vertigo VAS scores between the day-case and inpatient groups (Table 2).

### Crossover and readmission

Nine out of fourteen patients (64%) allocated to the day-case group were admitted to the ward after surgery for one (*n* = 8) or two (*n* = 1) nights. Reasons for this were postoperative nausea and vomiting (*n* = 1), drowsiness (*n* = 1), at request of the surgeon for longer observation time after intraoperative complications (gusher (without postoperative vertigo) *n* = 1, hypertension *n* = 1, type IV allergic reaction *n* = 1), late scheduled surgery (*n* = 2), social reasons (*n* = 1), or due to an unclear reason (*n* = 1). No patients required readmission.

## Discussion

This study allows for a comparison of QoL, hearing outcomes, patient satisfaction, (re)admission rates and complications between inpatient and day-case cochlear implantation in adults. We found an equal QoL in both groups (mean difference = 0.09 points) at three weeks postoperatively. At 1 year postoperatively the scores differed 0.17 points, however, at this follow-up moment the number of patients that fulfilled this questionnaire did not match the sample size calculation. The HUI3 hearing subscore was statistically significantly higher (0.08 points) but within the equivalence margin for the inpatient group at 3 weeks postoperatively and not significantly different (0.03 points) at 1-year follow-up, and therefore not considered to be clinically relevant. Over the follow-up period of 1 year, we found that there were no significant differences between the inpatient and day-case group regarding patient satisfaction, subjective and objective hearing results, and complication rates, except for the physical health subscore of the GHSI. This score was significantly lower in the day-case group at 3 weeks (67 versus 39) and 1 year (83 versus 53) postoperatively. When looking at the baseline scores, the preoperative physical health subscore was already worse in the day-case group (60 versus 47) which could explain these findings. Furthermore, the difference does not match up with the results of the GBI. The GBI physical health subscores were not significantly different between groups postoperatively even though the three questions assessing the physical health are practically the same for both questionnaires [25, 33, 34]. Another interesting between-group difference was the significantly higher tinnitus burden scores for all three tinnitus questionnaires in the inpatient group compared to the day-case group at 1-year follow-up, which again could be influenced by the differences between groups at baseline and is unlikely to be explained by the difference in day-case or inpatient approach.

Regarding the feasibility of cochlear implantation in a day-case setting in terms of crossover, 64% of our patients allocated to the day-case group ended up staying overnight after surgery due to different causes. This admission rate is substantially higher than previously reported admission rates. In a systematic review of our research group [35] on this topic we found five studies reporting an admission rate ranging between of 0% to 15% following day-case cochlear implant surgery. An explanation for the difference found could be that four out of five of these articles [5–8] concerned a pediatric population and only one study assessed day-case surgery in adult patients [10], reporting an admission rate of 0% in 50 patients. Another potential cause of our high admission rates may be that all patients were required to stay for a four-hour observation period in the inpatient ward postoperatively, making the decision to stay overnight easier for both patient and surgeon. The alternative, and our current daily practice, is to admit patients to a day-case unit, possibly changing the mindset of both patient and surgeon. Lazard et al. [36] found that after introducing major otologic surgery in a day-case setting, their crossover rates from day-case to inpatient care decreased over the course of 3 years from 10 to 21%, to 1%. The increase of feasibility of day-case surgery was related to better preoperative planning, scheduling, patient selection and an increase of experience with day-case surgery of the involved medical personnel (surgeon, anesthesiologist and paramedical team).

For the current study we tried to limit patient selection as much as possible up front. Two of our inclusion criteria regarded the feasibility of a day-case approach: general health allowing general anesthesia in an outpatient setting as assessed by an anesthesiologist and quick access to communication and transportation in case of any complications. Besides this, our hospitals’ protocol prohibits patients to be alone the first postoperative night. If this is the case patients are admitted for the first postoperative night. Despite selecting patients in this way, 64% of day-case patients had to stay overnight. Only two patients, suffering from postoperative drowsiness and nausea with vomiting, were too sick to be discharged. Three of our nine crossover patients were due to intraoperative complications with an indication for longer postoperative observation. One patient happened to have no transport arranged and two patients, who also happened to be of age (77 and 81 years old) arrived back at the ward around 5:30 pm due to late scheduled surgery, possibly influencing the decision for an overnight stay for the convenience of the patient. Only one patient stayed without a clear reason reported, the only potential reason could be the one-hour travel distance to her home. Thereby, three out of nine crossovers could have been prevented with better surgical planning (time of surgery) and better preoperative counseling concerning postoperative transportation. We did not see a relation between crossover and the surgical approach (suprameatal approach versus post tympanotomy), insertion site (round window versus cochleostomy), type of anesthetic used and the use of morphine.

When assessing the feasibility of day-case cochlear implantation in terms of safety we found no significant differences in complication rates between the inpatient and day-case group. Moreover, this is not to be expected given that the surgical procedure is the same for both groups and the occurrence of postoperative complications is not likely to be affected by the duration of postoperative stay. Teschner et al. [10] compared cochlear implant surgery between two clinics in Germany (four-day hospital stay postoperatively) and the United States (day-case) and found only a small difference regarding minor complications (including edema, hematoma, pain, nausea, vertigo, altered taste) without apparent explanation of the relation to the length of hospital stay, and no significant difference in the rate of major complications. Heilbronn et al. [37] reported a postoperative admission and emergency department visit rate after cochlear implant surgery of 6.9% (100 of 1444 patients).

Besides the feasibility, the desirability of a day-case approach for the patient should be taken into account. We assessed the patient satisfaction and found that patients in both groups experienced their first postoperative night equally easy. Furthermore, 75% of patients allocated to the day-case group would undergo the surgery in day-case again. Only two patients felt more anxious because the surgery was planned in a day-case setting. However, when assessing the satisfaction of patients in the inpatient group, 86% found it pleasant to have spent the night in the hospital and 93% would not have preferred to have spent the night at home after surgery. Only one study [5] assessed patient/parental satisfaction specifically regarding day-case (pediatric) cochlear implantation and found an overall satisfaction with day-case surgery of 91%. Preoperative anxiety was present in 34% of cases and 44% would choose a day-case approach again, compared to 19% that would prefer surgery in an inpatient setting. Tysome et al. [12], evaluating patient satisfaction after major ear surgery, found that inpatient and day-case patients were equally happy with their length of hospital stay. They also found that a night in the hospital did not enhance patients’ satisfaction.

Another interesting consideration is the effect on health care related costs of a day-case approach of cochlear implantation. This is data that we have acquired as part of our randomized controlled trial [19]. However, due to the extent of this data the cost evaluation will be analyzed in a separate manuscript.

In interpreting our findings, the following considerations need to be taken into account. A limitation of this trial is that inclusion was only possible for patients with good understanding of the Dutch language and if they had quick access to communication and transportation in case of any complications. Secondly, our protocol reads that we planned a sensitivity analysis using data acquired from patients that opted not to be included in the study [19]. Unfortunately, only three patients were willing to fulfill the questionnaires without participating in the randomization process. Therefore, we decided not to perform a sensitivity analysis and as a result, the generalizability of this study was not assessed. Also, crossover from the inpatient group to the day-case group was not encouraged, therefore, it is not known how many patients allocated to the inpatient group would have been capable of same day discharge. A final limitation concerning the used outcome measures is that the TQ, THI and DHI questionnaire, although often used to study outcomes, are not validated tools for assessment of treatment outcome.

## Conclusion

Overall, when taking QoL, patient satisfaction, objective and subjective hearing outcomes and postoperative complications into account, a day-case approach of unilateral cochlear implantation showed no clinically relevant postoperative differences on listed outcomes with inpatient patients. The ideal conditions for day-case surgery are based on deliberate patient selection, surgical planning with enough postoperative time for observation and recovery, and the mindset of a day-case approach of both patient and the surgical team. To increase patient satisfaction regarding day-case surgery attention must be paid to the provision of preoperative information and controlling postoperative pain.

### Supplementary Information

Below is the link to the electronic supplementary material.

Supplementary file1 (PDF 315 KB)

Supplementary file2 (PDF 85 KB)

Supplementary file3 (PDF 82 KB)

## Data Availability

The data is not available.
