# Supplementary material for: Quality of life, hearing results, patient satisfaction and postoperative complications of day-case versus inpatient unilateral cochlear implantation in adults: a randomized controlled, equivalence trial
Source: Eur Arch Otorhinolaryngol. 2024 Jan 5;281(5):2313–25. doi: 10.1007/s00405-023-08352-8 (PMC11023990; doi:10.1007/s00405-023-08352-8)
Supplement: Supplementary file 3 — Supplementary file3 (PDF 82 KB) [file 405_2023_8352_MOESM3_ESM.pdf]

Appendix 3. Utrecht Patient Satisfaction Survey

	**Inpatient (n=14)**	**Day-case (n=12)**	**Difference (95% CI)**
Q1 (Did you feel more anxious because the surgery was planned in an inpatient/a day-case setting?) (n (%))<br>*Yes*	0 (0)	2 (17)	-17% (-40 to 9)*
Q2 (Did you feel less anxious because the surgery was planned in an inpatient/a day-case setting?) (n (%))<br>*Yes*	5 (36)	2 (17)	19% (-16 to 48)*
Q3 (Did you find it pleasant that you had/did not have to spend the night in the hospital after the surgery?) (n (%))<br>*Yes*	12 (86)	10 (83)	2% (-26 to 31)*
Q4 (If you would have the choice: would you undergo the surgery in an inpatient/day-case setting again next time?) (n (%))<br>*Yes*	12 (86)	9 (75)	11% (-21 to 40)*
Q5 (Would you have preferred to have spent the night at home/in the hospital after the surgery?) (n (%))<br>*Yes*	1 (7)	6 (50)	**-43% (-68 to -7)***
Q6 (Were you content with the hospital admittance in general?) (n (%))<br>*Yes*	14 (100)	11 (92)	8% (-14 to 30)*
Q7 (How easy or difficult was the first night after the operation on a scale from 0 to 10?) (mean (SD))	3.5 (2.3)	4.9 (2.3)	-1.4 (-3.3 to 0.4)**

Group differences printed in bold were statistically significant (p<0.05) using the Fisher's Exact Test (2-sided)* or the Independent-Samples Mann-Whitney U Test**
